# Effects of Providing High-Fat versus High-Carbohydrate Meals on Daily and Postprandial Physical Activity and Glucose Patterns: a Randomised Controlled Trial

**DOI:** 10.3390/nu10050557

**Published:** 2018-04-30

**Authors:** Evelyn B. Parr, Brooke L. Devlin, Marcus J. Callahan, Bridget E. Radford, Jennifer M. Blankenship, David W. Dunstan, John A. Hawley

**Affiliations:** 1 Exercise and Nutrition Research Program, Mary MacKillop Institute for Health Research, Australian Catholic University, Melbourne, 3000 VIC, Australia; brooke.devlin@acu.edu.au (B.L.D.); marcus.callahan@myacu.edu.au (M.J.C.); bridget.radford@acu.edu.au (B.E.R.); david.dunstan@baker.edu.au (D.W.D.); john.hawley@acu.edu.au (J.A.H.); 2 Division of Endocrinology, Metabolism, and Diabetes, Center for Human Nutrition, School of Medicine, University of Colorado Denver, Denver, CO 80204, USA; Jennifer.blankenship@ucdenver.edu; 3 Baker Heart and Diabetes Institute, Melbourne, 3004 VIC, Australia

**Keywords:** diet, activity, sedentary, glycaemic control

## Abstract

We determined the effects of altering meal timing and diet composition on temporal glucose homeostasis and physical activity measures. Eight sedentary, overweight/obese men (mean ± SD, age: 36 ± 4 years; BMI: 29.8 ± 1.8 kg/m^2^) completed two × 12-day (12-d) measurement periods, including a 7-d habitual period, and then 5 d of each diet (high-fat diet [HFD]: 67:15:18% fat:carbohydrate:protein versus high-carbohydrate diet [HCD]: 67:15:18% carbohydrate:fat:protein) of three meals/d at ±30 min of 0800 h, 1230 h, and 1800 h, in a randomised order with an 8-d washout. Energy intake (EI), the timing of meal consumption, blood glucose regulation (continuous glucose monitor system (CGMS)), and activity patterns (accelerometer and inclinometer) were assessed across each 12-d period. Meal provision did not alter the patterns of reduced physical activity, and increased sedentary behaviour following dinner, compared with following breakfast and lunch. The HCD increased peak (+1.6 mmol/L, *p* < 0.001), mean (+0.5 mmol/L, *p* = 0.001), and total area under the curve (+670 mmol/L/min, *p* = 0.001), as well as 3-h postprandial meal glucose concentrations (all *p* < 0.001) compared with the HFD. In overweight/obese males, the provision of meals did not alter physical activity patterns, but did affect glycaemic control. Greater emphasis on meal timing and composition is required in diet and/or behaviour intervention studies to ensure relevance to real-world behaviours.

## 1. Introduction

In studies measuring the effect of dietary interventions on glycaemic control, the provision of meals increases scientific rigor and ensures that energy and nutrient intake are controlled. However, the provision of food may alter physical activity patterns by reducing incidental activity surrounding mealtimes as the need to obtain, prepare, or cook food is removed [1]. Whilst food provision, including the specific timing of an individual’s dietary intake, is integral to assessing physiological outcomes and responses, it is unknown whether changing meal times through the provision of food over an entire day influences an individual’s blood glucose regulation. It is well-known that the dietary intake of carbohydrate has the greatest effect on glycaemic control. Dietary patterns where carbohydrate intake is replaced by dietary fat intake have become of interest [2,3] in response to the increasing rates of type 2 diabetes diagnosis worldwide [4]. Therefore, investigating the effects of a high-fat compared with a high-carbohydrate diet, when also considering the habitual dietary intakes, in individuals at risk of developing type 2 diabetes is of interest.

Physical activity and voluntary movement in the 3-h postprandial meal periods assists with daily blood glucose regulation and minimises the postprandial and nocturnal excursions that contribute to prediabetes risk [5]. For individuals with poor blood glucose control, sedentary behaviour (i.e., prolonged sitting) is highly prevalent, and has been shown to negatively impact blood glucose in the postprandial meal periods compared with when short bouts of activity are performed [6]. Although studies of temporal inactivity have demonstrated an increase in sedentary behaviour across a day [5,7,8], there is a lack of evidence on the extent to which the provision of meals of differing compositions affects activity patterns throughout a day and, in turn, glycaemic control in postprandial periods. Furthermore, an understanding of the timing of meal consumption has been proposed to be as important as the energy content or macronutrient composition of the diet in improving health status [9,10].

While meal frequency and later eating occasions have been linked to increased obesity [11,12], the timing of eating occasions is especially important for blood glucose regulation [13]. The assessment of dietary intake has progressed to technology-based methods that reduce the well-known limitations of inherent measurement error and bias in written recordings [14,15]. Recent studies have started to use methods such as smart-phone applications [16,17], wearable cameras [18], and online methodologies [19] to reduce participant burden and obtain accurate dietary intake records, as well as ensure that the data collected is prospective rather than retrospective. Many current methods of collecting dietary information may not achieve the accuracy that is required to analyse the timing or patterns of energy intake, and necessitate the use of novel technology-based methodologies to collect such information. Recently, the spread and timing of energy intake throughout the day using an iPhone application in adults highlighted the “back-ended” nature of energy intake, as well as the large number of eating occasions throughout a day [16]. Therefore, from the perspective of understanding physiological mechanisms, there is likely to be a mismatch in controlled dietary studies that cannot replicate the uneven/atypical spread of meals, making the interpretation of such results less applicable to real-world scenarios.

The purpose of the present study was fourfold: (1) to assess whether the provision of food in a dietary intervention study altered physical activity patterns compared to a habitual period and between different diets of different composition; (2) to determine the effect of meal composition (high-fat versus high-carbohydrate) on postprandial glucose responses compared with baseline habitual dietary intake; (3) to quantify and compare meal-to-meal postprandial glucose responses and activity patterns between dietary interventions; and (4) to explore novel dietary assessment analyses to obtain accurate dietary intake data, including timing and patterns of intake. It was hypothesised that: (1) the provision of food would not alter total daily activity, but as a consequence of reduced meal preparation may reduce the activity patterns in the preprandial and postprandial meal periods; (2) that a high carbohydrate diet would impair glycaemic control compared with a high-fat diet and participants’ habitual diet; and that (3) due to the expected reduction in physical activity and increased sedentary time, there would be greater postprandial glycaemic excursions in blood glucose during the high carbohydrate diet compared with the high fat diet, with the greatest postprandial response in the evening.

## 2. Materials and Methods

### 2.1. Participants and Study Design

Overweight/obese men (body mass index (BMI) 27–32.5 kg/m^2^) aged 30–45 years and with a sedentary lifestyle (in terms of both activity and job; <150 min/week of moderate-intensity exercise, for >3 months and >3 h/d sitting) were recruited. Telephone pre-screening compromised medical history, age, height, and weight (in order to calculate BMI; using weight (kg)/height (cm^2^)), weight history, and screening of dietary habits. Exclusion criteria were: major or chronic illness (i.e., diabetes, cardiovascular disease), illness that impairs mobility or eating/digestion, previous bariatric surgery, shift workers, smokers, individuals with strict dietary intake regimes (i.e., vegan, avoidances of principal study foods), individuals who were restricting their dietary intake (i.e., actively trying to lose weight), and individuals who had not been weight stable for the last three months. Due to several reports highlighting the effects of temporal eating patterns on physiological measures [19,20], individuals who self-reported not regularly consuming a breakfast meal or who did not report having a regular consumption of three main meals per day (i.e., breakfast, lunch, and dinner) were also excluded.

The study was undertaken at the St Patrick’s (Fitzroy, Victoria, Australia) campus of Australian Catholic University between September 2016 and February 2017. The study was approved by the Human Research Ethics Committee of the Australian Catholic University (2016-77H), was registered with the Australian New Zealand Clinical Trials Registry (ACTRN12616000637448), and all of the participants provided written informed consent. In a randomised crossover study design, each participant completed two experimental conditions with an eight-day (8-d) washout period between the conditions, and a 7-d habitual monitored period prior to each condition. Randomisation was determined using computer generated random numbers and sealed opaque envelopes (block-randomisation, *n* = 4) that were revealed to study personnel after written consent; baseline body composition and resting energy expenditure (REE) were also obtained and measured from each participant in order to allow sufficient time to prepare food packages.

### 2.2. Experimental Conditions

Participants completed 7 d of habitual monitoring prior to each experimental condition. Throughout the 7 d, dietary intake and timing were recording, interstitial glucose was measured, and activity monitors were worn (as detailed below). The two dietary conditions tested were (A) a high-fat, low-carbohydrate diet (HFD) for five days consisting of 67% of total energy intake (TEI) from fat, 15% from carbohydrate (CHO), and 18% from protein; and (B) an isocaloric high-carbohydrate, low-fat diet (HCD) comprising 67% of TEI from fat, 15% from CHO, and 18% from protein (Table 1). The nutrient composition of all of the foods in each experimental condition was obtained using FoodWorks© (Version 8, Xyris Software, Brisbane, Australia). Total daily estimated energy intake (kJ/d) was calculated using REE measured at the first baseline visit × 1.4 activity factor. Energy distribution was evenly spread over the day with 33.3% of energy provided at each of the three main meals (breakfast, lunch, and dinner; see Supplementary Table S1). No snacks were provided or consumed during each of the 5-d experimental periods. Each of the meals that were provided matched the composition of the two dietary conditions (HFD and HCD), but the foods provided varied to increase ecological validity. Participants were instructed to consume the meals at standardised times throughout both experimental conditions (within ±30 min of 0800, 1300, and 1830 h). All of the food during each of the 5-d dietary experimental diets was provided to participants, and daily food checklists were completed to maximise compliance. During both 5-d experimental dietary conditions, participants were instructed to abstain from alcohol, whilst habitual caffeine consumers were instructed to consume and record caffeine intake (no added milk or sugar/sweetener) as usual on all of the days. Participants were able to consume water ad libitum, and were instructed to record daily water intake.

### 2.3. Study Protocol

**Figure 1:**
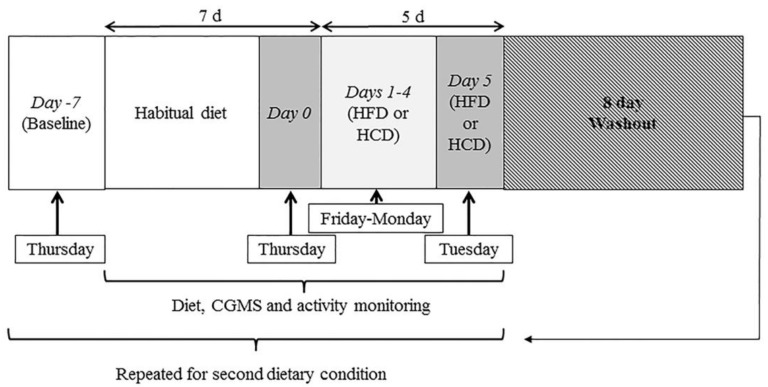
Study design schematic. Participants (*n* = 8) visited the laboratory on 10 occasions. Two trial conditions (high-fat diet (HFD; 67% total energy intake (TEI) fat, 15% TEI carbohydrate, and 18% TEI protein) and high-carbohydrate diet (HCD; 67% TEI carbohydrate, 15% TEI fat, and 18% TEI protein) were completed in a randomised order separated by an 8-d washout period. Dietary assessment, continuous glucose monitoring system (CGMS), and activity monitors were all worn and assessed throughout the 7-d habitual and 5-d experimental periods (total 12 d) for each trial condition.

Participant measures were conducted at the laboratory on Day 0 (end of 7-d habitual period) and on Day 5 (end of experimental dietary condition; as shown in Figure 1).

Day −7 (Baseline habitual period; Thursday): Participants arrived fasted (>10 h) at 0630 h for baseline measures before commencing their 7-d ‘habitual’ baseline recording period. REE was assessed for the final 15 min of 25 min using a calibrated TrueOne metabolic cart (TrueOneRMR, Parvo Medic, Sandy, UT, USA) to measure oxygen consumption (VO_2_), carbon dioxide (VCO_2_), and energy expenditure (kJ/d), with a mean coefficient of variation of 4%. Body composition was assessed via dual-energy x-ray absorptiometry (DXA; GE Lunar iDXA Pro, enCORE software Version 16, General Electric, Boston, MA, USA) to obtain total mass (kg), fat mass (FM; kg), fat-free mass (FFM; kg), bone mineral content (BMC; kg) and estimations of visceral adipose tissue (kg) under standardised conditions [21], with a CV of <1.5%. Waist, hip, and neck circumferences were measured using a metal tape measure in duplicate to the nearest 0.1 cm, or triplicate if the difference between the first two measures was greater than 0.5 cm. Following these measures, a continuous glucose monitor system (CGMS; iPro2 CGM with Enlite Sensor, Medtronic, Northridge, CA, USA) was inserted into the subcutaneous fat tissue of the lower back and secured with waterproof dressings. Following insertion, a one-hour period was used to allow the CGM sensor to adjust to the interstitial fluid before the initial calibration. A hand-held, commercial, time-stamped glucometer (Accu-Chek Performa II, Roche Diagnostics Ltd., Basel, Switzerland) was used for CGMS calibration from finger stick samples four times per day of wear (before each meal and sleep).

Day 0 (end of habitual period; Thursday): Participants arrived at 0630 h after an overnight fast for baseline measurements. Initially, the CGMS was replaced with a new sensor and monitor to ensure ongoing glucose monitoring. Participants then underwent REE and DXA measures as per Day −7 procedures. Participants consumed a provided habitual breakfast (details below) within ±30 min of 0800 h, before leaving the laboratory with a provided habitual lunch to be consumed within ±30 min of 1300 h. Participants returned to the laboratory at ~1830 h, rested for 5 min before blood pressure was measured prior to consuming their habitual evening meal. All of the pre-packaged food and fluid for the experimental condition (HFD or HCD) was then provided to participants for Days 1–4.

Days 1–4 (experimental period: Friday–Monday): Participants consumed pre-packaged and weighed meals (breakfast, lunch, and dinner) for Days 1–4 in their home/work environment.

Day 5 (end of experimental period; Tuesday): Participants arrived fasted at ~0630 h to complete all of the procedures as per Day 0, including REE, DXA, and breakfast consumption. Participants left the laboratory with a provided lunch meal according to the experimental conditions, and returned at ~1830 h for the evening meal. Participants removed all of the monitors the following morning (Day 6).

The Day −7 to Day 5 measurement period was then repeated in the same manner for the second experimental condition, following an 8-d washout period.

### 2.4. Dietary Control and Adherence

Prior to each experimental condition, participants were asked to record all food and fluid consumed over a 7-d period (habitual baseline, including two weekend days; Day −7 through to Day −1) using an innovative method to obtain habitual dietary intake behaviour. A study iPhone loaded with the Easy Diet Diary application (EDDapp; see Ambrosini et al., [22] for details) was provided to each participant to record all of the consumed food and fluid. In addition, participants were asked to use the iPhone to take images prior to and following meal consumption to provide an indication of the number of eating occasions, the time of the meal/snack, and the duration of the ‘eating time’, as each image provided a time stamp. Extensive written and verbal instructions were provided to participants by the study dietician to ensure that accurate records were obtained. A print-out summary of the foods consumed during the first 7-d baseline period was provided to participants, and they were asked to replicate the foods/meals that were consumed as closely as possible in the habitual period of their second experimental condition.

Participants were provided with all of the food/fluid consumed on Day 0. Dietary intake data recorded on the first three days of the 7-d baseline recording period was used to inform the food provided on Day 0 to replicate ‘habitual intake’. Specifically, the macronutrient composition matched participant’s recorded habitual intake for this 3-d period, while the total energy intake (TEI) provided was according to REE × 1.4 activity factor, and three meals were provided (33.3% of energy requirements provided at each meal). The types of foods provided were kept as consistent as possible with habitual food items. The d-0 food we provided was matched between both experimental conditions. Food for the experimental dietary interventions (HFD and HCD) and Day 0 was either pre-prepared (Dineamic, Camberwell, VIC, Australia) or purchased from the local supermarket and weighed and packed for each individual participant in labelled bags for each day (Days 1–5) and meal (breakfast, lunch, and dinner). Participants were provided with a comprehensive study handbook to record finger prick blood glucose sample results, sleep details, activity monitor removals, record dietary intake during baseline periods, and complete a food checklist for both experimental dietary conditions.

### 2.5. Activity Monitoring

Participants were asked to continue their regular daily activities (i.e., work and home life) throughout the study period. Throughout each 7-d habitual and 5-d experimental periods (12-d total), participants wore an activPAL inclinometer (activPAL3TMtri-axial physical activity monitor, PAL-technologies Ltd., Glasgow, Scotland) on the thigh, and an ActiGraph accelerometer (ActiGraph GTX3+, Pensacola, FL, USA; during waking hours only) worn around the waist over the right hip, to assess and monitor physical activity and movement patterns. Participants were asked to continue with their habitual sleep routine and sleeping patterns throughout the trial, whilst a SenseWear armband (SWA; Bodymedia, Pittsburgh, PA, USA) was worn on the tricep muscle continuously throughout the 12-d habitual and experimental periods for estimates of energy expenditure.

### 2.6. Data Analysis

Habitual dietary intake of all of the foods and beverages reported throughout the baseline recording periods was analysed using FoodWorks© (Version 8, Xyris, Brisbane, QLD, Australia) to estimate nutrient composition. Vitamin and mineral supplements were excluded. Average energy, macronutrient (CHO, protein, fat, and alcohol), and fibre were obtained. Images taken before and after food/fluid consumption were assessed, and the time of the photo was recorded. The number of eating occasions over a 24-h period and the ‘eating time’ (minutes between the before and after images) were recorded. An eating occasion was defined as an eating/drinking episode providing at least 210 kJ, and a minimum of 15-min elapsed between occasions [11]. Self-designated descriptions of what the main meal was (i.e., breakfast, lunch, and dinner) by each participant were used, and any snacks were designated to morning, afternoon, or evening snacks based on the time relative to the main meals. Where the after-eating images were not recorded, an average eating time of 15 min was used. Due to technical problems, the habitual dietary intake of one participant was not obtained; therefore, dietary analysis was performed on *n* = 7.

The glycaemic index (GI) and subsequent glycaemic load (GL) of each meal provided in the experimental period was estimated using data from the International GI database [23]. As most of the foods had multiple GI values, the selection of the GI was made hierarchically in order of preference from: (1) the same brand and method of preparation; (2) an Australian tested food; (3) an average value; or (4) the closest match (e.g., matched to food item with similar amount of CHO) [24]. The percentage of available CHO (excluding fibre) that each food item contributed to the total meal was multiplied by the GI value. Values were summed to estimate the GI of the total meal. The formula used for calculating the GI of a meal using the GI values of the individual food items was:

Meal GI = {(GI food A × (CHO avail. food A g/CHO meal avail. g) + (GI food B × (CHO avail. food B g/CHO meal avail. g) + …)}

The GL was calculated for each meal for each participant by multiplying the GI of the meal by the amount of CHO available, which was specific to the allocated calorie band based on baseline tests, and dividing by 100. The formula for calculating GL of a meal was:

Meal GL = (GI meal × CHO available g meal)/100

Twenty-four h CGMS data from midnight to midnight was used to calculate mean, peak, and fasting glucose (60 min prior to self-reported wake time) and area under the curve (total AUC; trapezoid method with a baseline of 0 (using GraphPad Prism 7.01, GraphPad Software Inc., CA, USA)), for each habitual day (Day −6 to Day −1) compared with the experimental days (Days 1 to 5), and averaged for each period. Day −7 and Day 0 were not included in the analysis due to inserting and changing the sensor, which reduced the total sensor time (<24 h). Due to issues with sensor detachment for one participant, CGMS analysis was performed on *n* = 7. Using the meal timing data, the CGMS 3-h postprandial periods were analysed from the reported time of finished meal consumption for all of the habitual days and, using the average meal consumption time, the estimated time of finished meal consumption for the experimental days. Postprandial CGMS measures of pre-meal (mean glucose 15 min prior to start of meal), mean, peak, and postprandial glucose (3 h post-meal glucose), total AUCmeal (trapezoid method with baseline of 0), and iAUCmeal (trapezoid method with pre-meal glucose as baseline) were then calculated.

Analysis of all of the activity monitoring was performed using SAS 9.4 (SAS Institute, Cary, NC, USA). The 1-min epoch data files from ActiGraph accelerometers were processed to derive average sedentary (<100 counts per min (cpm)), light-intensity (100–1951 cpm), and moderate–vigorous intensity (≥1952 cpm) activity time on valid (≥10 h) days [25]. Similarly, the time spent sitting, time spent sitting for ≥30 min, standing, and stepping were estimated from activPAL data for the waking periods (from self-reported sleep and waking times) of the habitual and experimental days, on valid (≥10 h) days. SWA generated estimates of daily energy expenditure (EE) were measured through the recorded data based on the SWA proprietary algorithm, as previously validated [26], using SenseWear Professional 7.0 software (Bodymedia Inc., Pittsburgh, PA, USA). The resulting Excel files were analysed for energy expenditure during waking hours and sleeping hours.

### 2.7. Statistical Analysis

Statistical analyses were performed using SPSS (Version 22, SPSS Inc., Chicago, IL, USA). Data from the two conditions was analysed using linear mixed models (LMM), for changes across time (habitual versus experimental) and between conditions. All of the models were adjusted for potential covariates explaining residual outcome variance (age and BMI) and period effects (trial order). When significance was observed, post-hoc comparisons between conditions were conducted within the LMM based on the least significant squares (LSD) test. Significance was set at *p* < 0.05, and all of the data are presented as mean ± SD, with mean differences and 95% confidence intervals for differences when significant. The sample size for this investigation was based on the primary outcome measures of metabolomics from serum and skeletal muscle samples. As such, all of the outcomes presented in the current manuscript are secondary, as listed on the clinical trial registration.

## 3. Results

**Figure 2:**
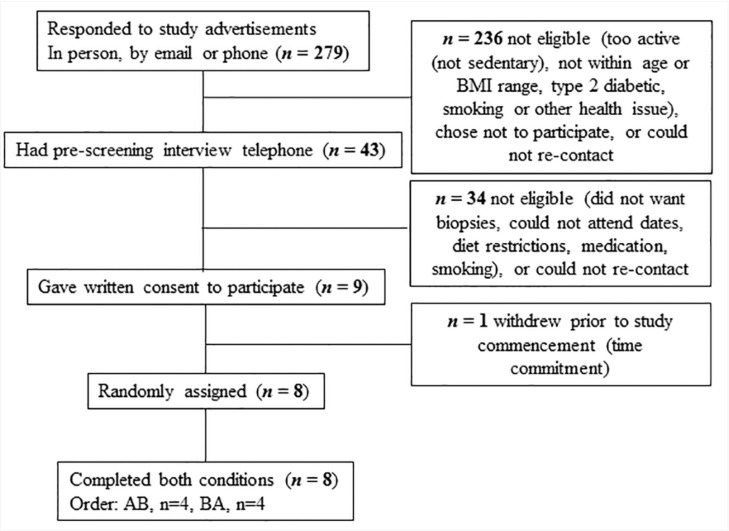
Consolidated Standards of Reporting Trials (CONSORT) flow diagram of participant recruitment.

Of the 43 participants who were phone-screened, nine eligible participants consented, and eight participants (mean ± SD, age: 36 ± 4 years; BMI: 29.8 ± 1.8 kg/m^2^; BM: 97.2 ± 9.9 kg; body fat percentage: 33 ± 3%; waist-to-hip ratio (WHR): 0.96 ± 0.05) were randomised and completed both trial conditions (Figure 2). Baseline measurements of body composition and REE did not differ between conditions (Table S3). Furthermore, body composition and REE did not change across the habitual diet period, or with either experimental diet (Table S3). Although a main effect of condition was observed for fat and lean mass (*p* = 0.03 and *p* = 0.01, respectively), there were no within-condition effects.

Habitual dietary intake, assessed through the 7-d EDDapp and photo records, was not different between pre-HFD and pre-HCD weeks for total energy, carbohydrate, and total fat intakes, nor for the percentage of TEI of carbohydrate, protein, or fat (Table 1). However, prior to the HCD, participants consumed less protein (g) (−22 g, *p* < 0.05).

Participant’s EDDapp records and photos indicated 4 ± 1 eating occasions (>210 kcal) per day across both habitual periods. The incidence of energy intake consumption occurring within the 3 h postprandial periods of breakfast, lunch, and dinner was 55 ± 31%, 46 ± 28%, and 42 ± 28%, respectively, for both conditions. Participant compliance with taking photo records was 78% across both habitual periods. An average of 65 photos were taken over the 7 d, with fewer before-eating photos missed (i.e., reported consuming energy but no photo was taken; 10%) compared to after-eating photos (33%). Photos (before and/or after) of snacks were more frequently missed (35%) than photos of main meals (12%). A large proportion of both before (65%) and after (79%) photos of evening snacks (>15 min post dinner) were missed. When evening snack photos were missed, these days were excluded from the calculations of the eating window over the course of the day and the timing of energy intake before sleep.

Across both habitual periods, the average time spent eating was 16.5 ± 6.5 min, with a total eating window across the day of 10.7 ± 1.0 h from ~0820 to ~1940 h. From the available photos and times, the first eating occasion (from waking) was shorter in the habitual periods (1.3 ± 0.5 h) compared to in the experimental period (1.8 ± 0.4 h; main effect of time, *p* = 0.005) for both diets. Similarly, the last energy intake before sleep was shorter in the habitual period (3.0 ± 0.9 h) compared to the experimental period (3.7 ± 1.1 h; main effect of time, *p* = 0.048), with no effect of diet.

**Figure 3:**
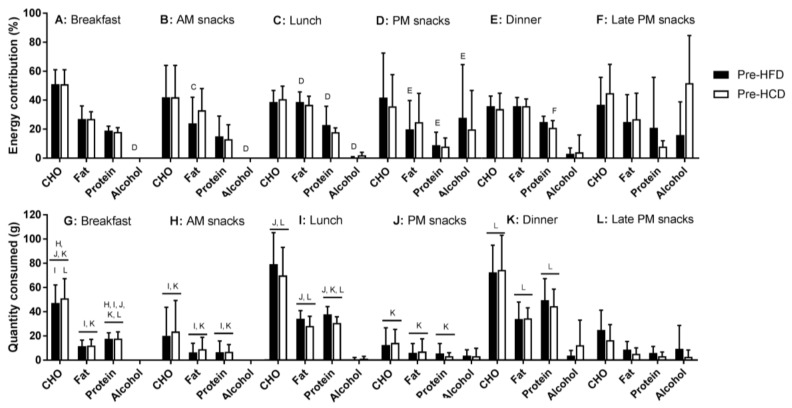
Proportion (A–F) and absolute (G–L) amounts of macronutrients consumed during the two 7-d habitual periods. Habitual diets were consumed prior to consuming the provided diet for sedentary males with overweight/obesity (*n* = 7). Key: Pre-HCD, prior to the high carbohydrate condition; Pre-HFD, prior to the high fat diet condition; Breakfast, lunch and dinner meals were denoted by the participant, with AM and PM snacks being caloric consumption in between the main meals and Late PM snacks being after (>15 min after) the dinner meal. Letters B-L denote significance (*p* < 0.05) between the associated letter time point within each dietary variable.

The percentage of energy reported to be consumed from breakfast, lunch, and dinner during the habitual periods was 15 ± 4%, 29 ± 6%, and 35 ± 10%, respectively, with no differences between the two habitual periods (Table S2). The remainder of the energy intake was consumed between breakfast and lunch (AM snacks; 6 ± 6%), lunch and dinner (PM snacks; 6 ± 5%), and after dinner (late PM snacks; 8 ± 6%). The macronutrient distribution was significantly different between meals and snacks for absolute amounts of carbohydrate, protein, and fat (main effect of meal: all *p* < 0.001; Figure 3 and Table S2), with the percentage of energy of each meal from fat and protein also differing between meals and snacks (main effect of meal: *p* = 0.03 and *p* = 0.04, respectively). However, the percentage of energy at each meal from carbohydrate was not different between meals (*p* = 0.22). The absolute protein intake (grams) was skewed higher at dinner, compared with lunch and breakfast, but there were no differences between the two habitual periods within meals.

For activity during waking-hour periods, no differences were observed between the activPAL and ActiGraph outputs of activity type (sedentary to moderate-vigorous physical activity (MVPA) and sitting to stepping) and step counts, with large proportions of time in waking hours spent sedentary (~59%, ~9.2 h/d) and sitting (~59%, 9.5 h/d), and limited time spent doing MVPA (~4%, 0.6 h/d (39 min/d)) or stepping (~12%, 2 h/d) (Table 2). No differences in activity measures were observed between habitual periods or between experimental conditions for 1-h preprandial or 3-h postprandial periods (Table 3). Preprandial activity patterns from ActiGraph monitors did not differ between lunch and dinner. However, prior to breakfast, a greater proportion of time was spent sedentary (60%; main effect of meal, *p* < 0.01; Table 3), compared with lunch and dinner. Postprandial activity patterns at dinner showed a greater proportion of sedentary behaviour (70%) compared with breakfast (57%) and lunch (60%), respectively, and a reduction in the number of steps taken (breakfast: 927 steps, lunch: 893 steps, dinner: 486 steps; Table 3).

For 24-h CGMS, no differences were observed between the two habitual periods. The HCD increased peak glucose (+0.8 mmol/L, 95%CI: 0.2–1.5 mmol/L; *p* = 0.01), compared to the habitual period (Table 2). Whilst fasting glucose did not change, consumption of the HCD elevated peak (+1.6 mmol/L, 95%CI: 1.0–2.2 mmol/L; *p* < 0.001), mean (+0.5 mmol/L, 95%CI: 0.2–0.7 mmol/L; *p* = 0.001), and total AUC glucose (+670 mmol/L/min, 95%CI: 300–1040 mmol/L/h; *p* = 0.001) compared with the HFD (Table 2).

No differences in CGMS measures were observed between the postprandial habitual meal periods (data not shown). However, the consumption of the HCD increased total AUCmeal mean and iAUCmeal glucose for all three meal periods, and increased peak glucose at lunch and dinner compared with the habitual period (Table 4). Conversely, the HFD consumption lowered peak and post-meal glucose at breakfast and pre-meal glucose at dinner, compared to the habitual period. In comparison, the HCD increased peak, mean, post-meal, total AUCmeal, and iAUCmeal glucose (all *p* < 0.001) compared with the HFD for all of the meals, and pre-meal glucose was greater for lunch and dinner for the HCD compared with HFD. An effect of meal for peak glucose (*p* = 0.03) and pre-meal glucose (*p* = 0.002) was measured, where lunch peak glucose was greater than breakfast and dinner in the HCD condition, and pre-meal glucose was lower at dinner than breakfast in the HFD condition. Further, a meal effect for iAUCmeal (*p* = 0.014) was observed, where the iAUCmeal was lower following breakfast compared with lunch and dinner, respectively, in the HCD condition only.

## 4. Discussion

This is the first study to show that the provision of meals to overweight/obese male participants did not alter activity patterns and sedentary behaviour across a day or in the time before and after meal consumption. There was a clear increase in sedentary behaviour in the post-dinner period in both habitual and experimental periods, which coincides with typical circadian patterns of reduced glucose tolerance and action of insulin [27]. A novel feature of the present investigation was the comparison of habitual and experimental activity and behaviour patterns specific to the preprandial and postprandial periods along with the comprehensive evaluation of habitual compared with experimental periods of postprandial activity in an uncontrolled ‘free-living’ environment. Previous investigations have systematically reduced the number of steps in healthy people over three weeks, and demonstrated impaired glucose tolerance [28], which has also been shown to occur with just five days of reduced physical activity [29]. In laboratory settings, Dunstan et al. have shown improved effects on glucose regulation by breaking up prolonged sedentary behaviour (sitting) with light walking or simple resistance exercise in a range of individuals [6,30,31]. However, we are not aware of another study that has performed a comprehensive analysis of postprandial habitual activity, which highlights how the temporal pattern of physical activity stalls in the evening, with concurrent increases in sedentary behaviour patterns. The increased sedentary behaviour in the evening highlights the unhealthy routine adopted by this cohort. The assessment of physical activity and sedentary behaviour is a new field providing fruitful and applicable data towards strategies to improve glycaemic control and overall health.

The finding that physical activity is markedly reduced in the evening, following dinner, is supported in temporal analyses [5,7]. However, habitual sedentary behaviour with concurrent measures of meal timing, meal content, and glycaemic control has not been systematically evaluated. When adding 30 min of physical activity in as separate 10-min blocks post-meals, the post-lunch and dinner periods have shown the biggest gains in activity temporally [5], due to sedentary behaviour being the highest in these periods. Further, Reynolds et al. [5] also demonstrated that targeting 10-min walks post-breakfast did not change the amount of physical activity in the hour post-breakfast. However, our 3-h postprandial analysis showed that physical activity patterns between breakfast and lunch were not different. The 3-h postprandial dinner period could therefore be a target for introducing greater activity, and therefore muscle contraction, in order to better regulate post-meal glucose and reduce the risk of developing type 2 diabetes.

The preprandial activity patterns we measured reflect the short time between waking and consuming the first meal. Considering the “dawn phenomenon” [32], delaying breakfast, as in a time-restricted feeding model [16], may be beneficial in avoiding the morning peak glucose that occurs in individuals with impaired glucose tolerance (prediabetes) or type 2 diabetes. In turn, delaying breakfast for individuals with prediabetes or type 2 diabetes could allow a greater accumulation of physical activity (steps), allowing for the uptake of glucose from the bloodstream through muscle contraction. Glucose tolerance tends to decline at the end of a day, due to the reduction in insulin sensitivity in a normal circadian pattern [27]. We did not observe a decline in glucose tolerance whereby no differences in peak glucose were measured between breakfast and dinner, with a greater peak at lunch. The greater glycaemic load at the lunch meal explains the peak glucose being highest following lunch. Further, despite the different energy contribution of meals in the habitual period, there were no differences in carbohydrate intake between meals, and therefore were no differences between meals for any glycaemic variable measured.

The smartphone app, Easy Diet Diary, in combination with taking photos before and after meals, was used to reduce burden in dietary recordings, increase compliance, and allow information on the timing and duration of energy intake to be obtained, as well as the number of eating occasions. The habitual dietary analysis showed carbohydrate intake (g) was overall lower at breakfast, due to the ~50% smaller energy intake at breakfast (~15%) compared with lunch and dinner. Whilst Australian-specific data for the composition of meals is limited, the different distribution of macronutrients at each meal was expected due to the typical foods consumed at each meal, as evident in other countries [33]. The high-fat, low-carbohydrate (15% TEI from CHO, ~116 g CHO) diet only improved peak and post-meal glucose at breakfast, compared with the habitual diet. Due to typical Australian breakfast foods being higher in carbohydrate and lower in protein, the GL was greater, and therefore the high-fat diet breakfast had a low GL (24 GL units), which contributed to the lowered peak and post-meal glucose concentrations. The high-carbohydrate diet contained carbohydrate (65% TEI, ~515 g) in amounts twice that of reported habitual intakes (~250 g) by participants. Consequently, the high-carbohydrate diet had a significant effect on all of the blood glucose measures, with the exception of pre-meal glucose concentrations. Future intervention studies that are aiming to replicate ‘real-world’ situations should optimise the nutritional intake to be as similar to temporal habitual intakes in terms of energy as well as macronutrient composition, whenever possible.

Typically, an increased eating frequency is associated with increased energy intake, as is the later timing of consumption of the last meal [34]. We found the majority of missed photos were later in the evening, which could have adversely affected the accuracy of interpreting our data for last meal consumption and sleep onset. The skewed and variable habitual dietary intake makes it difficult to replicate ‘real-life’ situations, and also highlights the unconscious coupling of food consumption with total amounts of activity [35]. Further, the methodology of using activity monitors to collect behavioural data provides limited information regarding the type of sedentary behaviour undertaken, such as television viewing, which has been shown to adversely affect energy intake [36].

The strengths of this study are in the high volume of wear time and number of days of activity monitoring, as well as greater accuracy and lower burden with the dietary collection technique. The crossover study design (within subjects), along with a monitored, non-invasive habitual period, has strengthened the exploratory findings. However, there are limitations to this investigative work, which include small sample size, a lack of glucose tolerance measures before and after the dietary interventions, and the high number of after-meal photos that were missed, leading to an inability to ascertain the time between the last energy intake and sleep. As the investigation was completed with male participants, it is unknown whether the same patterns would be evident in females. Lastly, we did not collect any information with regards to the type of sedentary behaviour (i.e., television viewing), which may affect energy intake patterns [36]. In future studies, strategies to reduce end-of-day sedentary behaviour should consider the coupling of specific dietary advice in order to best improve blood glucose regulation. Optimising glycaemic control is especially important as the risk of developing type 2 diabetes increases with age. Future interventions could also aim to establish optimal activity and diet for postprandial glucose control, especially with the impaired glucose sensitivity at the end of a day, which pairs with increased sedentary time.

## 5. Conclusions

In conclusions, the provision of experimental meals did not alter the total day or postprandial activity patterns of men who are overweight or obese and sedentary. Peak blood glucose was most affected by the high-carbohydrate diet, but the high-fat diet did not substantially alter blood glucose regulation compared with habitual intake. We observed increased sedentary activity across the day, without an associated change in peak and mean glucose, providing a clear target of the evening time established as the time for sedentary behaviour to be minimised.

## Figures and Tables

**Table 1:** Dietary intake breakdown of provided (habitual: *Day 0*; experimental: *Days 1–5*) and habitually consumed (habitual: *Day −7* to *Day −1*) diets for overweight, sedentary males (*n* = 7).

	Habitual Intake Pre-HFD (*Days* −*7* to *Day* −*1*)	Habitual Intake Pre-HCD (*Days* −*7* to *Day* −*1*)	Habitual Provided Intake (Day 0)	HFD (Days 1–5)	HCD (Days 1–5)
Energy (kJ)	11,021 ± 2328	10,483 ± 1953	11,839 ± 1565 ^1^	12,300 ± 1039	12,300 ± 1039
CHO (g)	259 ± 77	245 ± 56	322 ± 43	116 ± 10	515 ± 44
Sugars (g)	92 ± 41	89 ± 26	139 ± 37	31 ± 3	347 ± 29
CHO (% TEI)	38 ± 5	38 ± 8	44 ± 4	15 ± 0	67 ± 0
Protein (g)	125 ± 28	103 ± 21 *	117 ± 18	130 ± 11	130 ± 11
Protein (% TEI)	19 ± 2	18 ± 2	17 ± 2	18 ± 0	18 ± 0
Fat (g)	106 ± 19	94 ± 22	110 ± 22	223 ± 19	50 ± 4
Fat (% TEI)	36 ± 5	33 ± 4	34 ± 3	67 ± 0	15 ± 0
Saturated fat (g)	39 ± 7	37 ± 11	41 ± 10	114 ± 10	21 ± 2
Polyunsaturated fat (g)	15 ± 4	14 ± 3	19 ± 6	9 ± 1	6 ± 1
Monounsaturated fat (g)	39 ± 5	34 ± 9	36 ± 8	17 ± 2	13 ± 1
Fibre (g)	28 ± 5	24 ± 6	37 ± 10	29 ± 3	48 ± 4
Alcohol (g)	18 ± 24	16 ± 20	0 ± 0	0 ± 0	0 ± 0
Alcohol (% TEI)	6 ± 6	4 ± 5	0 ± 0	0 ± 0	0 ± 0

Key: HCD, high-carbohydrate diet; HFD, high-fat diet; CHO, carbohydrate; TEI: total energy intake. Data are mean ± SD; ^1^ Energy intake for provided meals (i.e., habitual Day 0 and HFD/HCD) was calculated based on resting energy expenditure (REE) × 1.4 activity factor for each participant. From linear mixed model (LMM) analysis, significantly different (*p* < 0.05); * compared to habitual pre-HFD.

**Table 2:** Activity and glucose monitor analysis across the habitual (*Day −7* to *Day 0*) and experimental (*Days 1–5*) periods for overweight, sedentary males (*n* = 8).

Variable	Habitual (*Day −7* to *Day 0*)		Experimental (*Days 1–5*)		*p*		
	Pre-HFD	Pre-HCD	HFD	HCD	Condition	Time	Condition × Time
CGMS ^1^							
Fasting glucose (mmol/L)	5.1 ± 0.3	5.1 ± 0.3	5.0 ± 0.4	5.2 ± 0.4	0.372	0.965	0.504
Peak glucose (mmol/L)	6.8 ± 0.3	7.0 ± 0.8	6.2 ± 0.2 ^†^	7.8 ± 0.9 ^#^	0.001	0.581	0.004
Mean glucose (mmol/L)	5.2 ± 0.3	5.4 ± 0.3	5.1 ± 0.3 ^†^	5.6 ± 0.3	0.002	0.868	0.081
Total AUC (mmol/L/min)	7520 ± 400	7751 ± 442	7288 ± 379 ^†^	7959 ± 416	0.002	0.924	0.095
activPAL ^2^							
Sitting (%)	58 ± 8	63 ± 9	58 ± 9	57 ± 15	0.422	0.213	0.190
Sitting for ≥30 min ^2^ (%)	25 ± 6	30 ± 10	25 ± 8	24 ± 13	0.453	0.160	0.148
Standing (%)	29 ± 8	26 ± 8	30 ± 9	30 ± 13	0.381	0.184	0.247
Stepping (%)	13 ± 3	18 ± 3	12 ± 3	13 ± 3	0.786	0.667	0.225
Steps (*n*)	4795 ± 1197	4642 ± 1389	4538 ± 990	4716 ± 1023	0.948	0.640	0.399
ActiGraph ^3^							
Sedentary PA (%)	58 ± 9	61 ± 9	61 ± 6	58 ± 12	0.620	0.620	0.115
Light PA (%)	37 ± 7	34 ± 8	35 ± 5	38 ± 11	0.454	0.378	0.086
Moderate–Vigorous PA (MVPA) (%)	5 ± 2	5 ± 2	4 ± 2	4 ± 2	0.641	0.171	0.876
Sensewear ^4^							
Energy expenditure, overall (kJ/d)	14,026 ± 2521	13,984 ± 2271	13,382 ± 1945	13,597 ± 2133	0.728	0.047	0.603
Energy expenditure, waking hours (kJ/d)	11,081 ± 2505	11,422 ± 2513	10,439 ± 1498	11,193 ± 2474	0.676	0.114	0.622
Energy expenditure, sleeping hours (kJ/d)	2568 ± 220	2560 ± 148	2398 ± 180	2523 ± 434	0.143	0.291	0.435

^1^ From iPro2 continuous glucose monitor systems (CGMS) analysed for 24 h from midnight to midnight, where the habitual period does not include Day 0 due to monitor changeover; ^2^ From activPAL and ActiGraph activity monitors where only waking hours were anlaysed, where total wake time was >10 h; ^3^ Sitting for >30 min periods is as a percentage of the time spent sitting; ^4^ From SenseWear Armband monitors estimated from total wear time (>98%) over 24 h. Data are mean ± SD; using LMM analysis, significantly different (*p* < 0.05); ^#^ compared to habitual within condition; ^†^ compared to HCD at same time. AUC: area under the curve; PA: physical activity.

**Table 3:** Activity monitor analysis from 1-h preprandial and 3-h postprandial periods across the habitual (*Day −6 to Day 0*) and experimental (*Days 1–5*) periods for overweight, sedentary males (*n* = 8).

Variable by Meal	Preprandial (1 h Pre Meals)				Postprandial (3 h Post Meals)			
	Habitual (*Day −6* to *Day 0*)		Experimental (*Days 1–5*)		Habitual (*Day −6* to *Day 0*)		Experimental (*Days 1–5*)	
	Pre-HFD	Pre-HCD	HFD	HCD	Pre-HFD	Pre-HCD	HFD	HCD
**Breakfast**								
Steps (*n*) ^1^	279 ± 126 ^L^	234 ± 102	171 ± 98 ^D^	211 ± 123 ^L^	941 ± 288 ^D^	1000 ± 534 ^D^	919 ± 335 ^D^	1033 ± 298 ^D^
SedPA (%) ^2^	60 ± 9 ^L^	68 ± 6 ^LD^	55 ± 7	61 ± 17 ^L^	58 ± 11 ^D^	57 ± 19 ^D^	57 ± 9 ^D^	56 ± 16 ^D^
Light PA (%) ^2^	35 ± 6 ^LD^	28 ± 5 ^LD^	42 ± 8	37 ± 15	37 ± 9 ^D^	36 ± 14 ^D^	38 ± 7 ^D^	39 ± 14 ^D^
MVPA (%) ^2^	5 ± 3	4 ± 4	3 ± 4	2 ± 4	5 ± 3 ^D^	6 ± 6 ^D^	5 ± 3 ^D^	5 ± 3
**Lunch**								
Steps (*n*)	429 ± 266	331 ± 153	287 ± 123 ^#^	425 ± 169	874 ± 458 ^D^	842 ± 381 ^D^	968 ± 377 ^D^	889 ± 318
SedPA (%)	49 ± 16	58 ± 16	57 ± 8	50 ± 16	60 ± 16 ^D^	61 ± 18 ^D^	59 ± 13 ^D^	59 ± 15 ^D^
Light PA (%)	43 ± 13	38 ± 14	39 ± 7	45 ± 14	36 ± 13	35 ± 17 ^D^	36 ± 10 ^D^	37 ± 14 ^D^
MVPA (%)	8 ± 6 ^#,D^	4 ± 2	3 ± 2	5 ± 5	5 ± 4 ^D^	4 ± 2	5 ± 4 ^D^	4 ± 2
**Dinner**								
Steps (*n*)	304 ± 164	333 ± 111	311 ± 167	338 ± 142	422 ± 236	413 ± 186	476 ± 253	634 ± 359
SedPA (%)	52 ± 12	52 ± 9	51 ± 13	53 ± 9	69 ± 8	71 ± 8	70 ± 8	69 ± 8
Light PA (%)	44 ± 9	43 ± 8	44 ± 10	45 ± 9	29 ± 7	26 ± 6	28 ± 7	28 ± 7
MVPA (%)	6 ± 2	5 ± 2	5 ± 5	3 ± 2	1 ± 2	3 ± 2	2 ± 2	3 ± 2

From ^1^ activPAL and ^2^ ActiGraph activity monitors where the 1 h pre- and 3 h post-meal periods were analysed as a percentage of valid wear time. MVPA, moderate vigorous physical activity. Data are mean ± SD; using LMM analysis, significantly different (*p* < 0.05); ^#^ compared to habitual within condition; ^L^ compared to lunch within trial; ^D^ compared to dinner within trial.

**Table 4:** Continuous glucose monitor analysis from 3-h postprandial periods across the average of the habitual periods (*Day −6 to Day −1*) and the two experimental diet (*Days 1–5*) periods from overweight, sedentary males (*n* = 7).

Glucose Measure	Condition			*p*		
	Habitual	HFD	HCD	Condition	Meal	Condition × Meal
Pre-meal glucose (mmol/L)						
Breakfast	5.2 ± 0.4	5.1 ± 0.3 ^D^	5.3 ± 0.4	<0.001	0.002	0.737
Lunch	5.1 ± 0.3	4.8 ± 0.2 ^†^	5.1 ± 0.5			
Dinner	5.1 ± 0.6	4.6 ± 0.5 ^†#^	5.1 ± 0.3			
Peak glucose (mmol/L)						
Breakfast	6.3 ± 0.7	5.7 ± 0.3 ^†#^	6.7 ± 0.7 ^L^	<0.001	0.014	0.339
Lunch	6.4 ± 0.6	5.9 ± 0.3 ^†^	7.5 ± 0.9 ^#^			
Dinner	6.1 ± 0.6	5.5 ± 0.5 ^†^	6.9 ± 0.6 ^#L^			
Mean glucose (mmol/L)						
Breakfast	5.5 ± 0.4	5.2 ± 0.3 ^†^	5.9 ± 0.5 ^#^	<0.001	0.251	0.709
Lunch	5.6 ± 0.4	5.3 ± 0.2 ^†^	6.2 ± 0.5 ^#^			
Dinner	5.4 ± 0.5	5.1 ± 0.5 ^†^	6.1 ± 0.4 ^#^			
Post-meal glucose (mmol/L)						
Breakfast	5.2 ± 0.6	4.9 ± 0.4 ^†#^	5.5 ± 0.7	<0.001	0.262	0.777
Lunch	5.4 ± 0.7	5.0 ± 0.4 ^†^	5.5 ± 0.6			
Dinner	5.4 ± 0.4	5.1 ± 0.4 ^†^	5.8 ± 0.4 ^#^			
Total AUC_meal_(mmol/L/min)						
Breakfast	1078 ± 89	1026 ± 60 ^†^	1162 ± 91 ^#^	<0.001	0.101	0.747
Lunch	1100 ± 78	1055 ± 43 ^†^	1235 ± 96 ^#^			
Dinner	1075 ± 98	1040 ± 42 ^†^	1208 ± 78 ^#^			
iAUC_meal_ (mmol/L/min)						
Breakfast	49 ± 52	26 ± 39 ^†^	121 ± 66 ^#,L,D^	<0.001	0.003	0.681
Lunch	96 ± 54	91 ± 36 ^†^	213 ± 157 ^#^			
Dinner	59 ± 84	83 ± 29 ^†^	197 ± 88 ^#^			

AUC, area under the curve; HCD, high carbohydrate diet; HFD, high fat diet. Data are mean ± SD; using LMM analysis, significantly different (*p* < 0.05) ^#^ compared to habitual; ^†^ compared to HCD at same meal; ^L^ compared to lunch; ^D^ compared to dinner.

## Supplementary Materials

The following are available online at http://www.mdpi.com/2072-6643/10/5/557/s1, Table S1: Dietary intake breakdown of the three meals for each of the two experimental diets (HFD and HCD) provided to participants; Table S2: Further dietary intake breakdown of the meals and snacks for each of the two Habitual periods for sedentary males with overweight/obesity (*n =* 7); Table S3: Anthropometric and resting energy expenditure measures from overweight, sedentary males (*n* = 8) from baseline, the end of the habitual period, and the end of each experimental dietary period, Table S4: Wear time for activity monitor analysis across the habitual (Day −7 to Day 0) and Experimental (Days 1–5) periods for overweight, sedentary males (*n* = 8); CONSORT statement.
