# Supplementary material for: Effects of Providing High-Fat versus High-Carbohydrate Meals on Daily and Postprandial Physical Activity and Glucose Patterns: a Randomised Controlled Trial
Source: Nutrients. 2018 Apr 30;10(5):557. doi: 10.3390/nu10050557 (PMC5986437; doi:10.3390/nu10050557)
Supplement: Supplementary file 1 [file nutrients-10-00557-s001.zip › S1 Online Supplementary Material.pdf]

Supplementary Online Information

Methods

Table S1. Dietary intake breakdown of the three meals for each of the two experimental diets (HFD and HCD) provided to participants.

	HFD (Days 1-5)			HCD (Days 1-5)		
	**Breakfast**	**Lunch**	**Dinner**	**Breakfast**	**Lunch**	**Dinner**
**Energy (kJ)**[2]	4072 ± 344	4093 ± 346	4140 ± 350	4088 ± 346	4069 ± 344	4232 ± 358
**CHO (g)**	38.7 ± 3.3	39.1 ± 3.3	38.7 ± 3.3	172.2 ± 14.6	170.7 ± 14.4	177.3 ± 15.0
**Sugars (g)**	5.3 ± 0.4	9.1 ± 0.8	16.3 ± 1.4	160.5 ± 13.6	96.8 ± 8.2	90.3 ± 7.6
**CHO (% TEI)**	15 ± 0	15 ± 0	15 ± 0	67 ± 0	67 ± 0	67 ± 0
**Glycemic index**	63.3 ± 0.0	52.7 ± 0.0	53.1 ± 0.0	47.5 ± 0.0	67.6 ± 0.0	51.0 ± 0.0
**Glycemic load**	24.5 ± 2.0	20.4 ± 1.6	19.7 ± 1.6	81.8 ± 6.6	115.0 ± 9.3	90.7 ± ±7.3
**Protein (g)**	42.2 ± 3.6	42.8 ± 3.6	43.5 ± 3.7	43.1 ± 3.6	43.6 ± 3.7	43.9 ± 3.7
**Protein (% TEI)**	18 ± 0	18 ± 0	18 ± 0	18 ± 0	18 ± 0	18 ± 0
**Fat (g)**	73.9 ± 6.2	74.1 ± 6.3	75.3 ± 6.4	16.3 ± 1.4	16.1 ± 1.4	17.6 ± 1.5
**Fat (% TEI)**	67 ± 0	67 ± 0	67 ± 0	15 ± 0	15 ± 0	15 ± 0
**Saturated fat (g)**	40.7 ± 3.4	45.2 ± 3.8	29.0 ± 2.5	8.9 ± 0.8	7.9 ± 0.7	2.8 ± 0.2
**Polyunsaturated fat (g)**	4.2 ± 0.4	2.3 ± 0.2	2.1 ± 0.2	2.2 ± 0.2	1.8 ± 0.1	1.3 ± 0.1
**Monounsaturated fat (g)**	11.6 ± 1.0	18.6 ± 1.6	20.9 ± 1.8	3.8 ± 0.3	4.2 ± 0.4	5.4 ± 0.5
**Fibre (g)**	8.1 ± 0.7	8..8 ± 0.7	12.2 ± 1.0	13.2 ± 1.1	13.2 ± 1.1	22.0 ± 1.9

Data are mean ± SD. CHO, carbohydrate; HCD, high carbohydrate diet; HFD, high fat diet; TEI, total energy intake.

Results

Table S2. Further dietary intake breakdown of the meals and snacks for each of the two Habitual periods for sedentary males with overweight/obesity (n-7).

	Habitual pre HFD (Days -7 to -1)						Habitual pre HCD (Days -7 to -1)					
	**B/fast**	**AM snacks[1]**	**Lunch**	**PM snacks[1]**	**Dinner**	**Late PM snacks[1]**	**B/fast**	**AM snacks[1]**	**Lunch**	**PM snacks[1]**	**Dinner**	**Late PM snacks[1]**
**Energy (kJ)**	1566 ± 368[b,c,d,e]	696 ± 839[c,e]	3294 ± 510[d,f]	633 ± 630[e]	3603 ± 1156[f]	1148 ± 1065	1693 ± 489[b,c,d,e,f]	857 ± 897[c,e]	2853 ± 569[d,e,f]	664 ± 765[e]	3805 ± 1128[f]	611 ± 403
**Energy (% of DEI)**	15 ± 3[b,c,d,e]	5 ± 6[c,e]	31 ± 8[d,f]	5 ± 5[e]	33 ± 10[f]	10 ± 8	16 ± 5[c,d,e,f]	7 ± 7[c,e]	27 ± 3[d,e,f]	6 ± 7[e]	37 ± 11[f]	6 ± 4
**Sugars (g)**	15.3 ± 6.3	12.1 ± 16.6	21.8 ± 13.9[d]	6.5 ± 9.5	22.2 ± 8.4	12.7 ± 10.8	19.8 ± 8.3[d]	11.4 ± 11.3[e]	16.5 ± 6.5	6.7 ± 5.1[e]	23.8 ± 17.3	12.6 ± 9.8
**Saturated fat (g)**	5.0 ± 2.0[c,e]	3.0 ± 4.0[c,e]	12.7 ± 3.4[d,f]	2.5 ± 3.4[e]	11.4 ± 4.7[f]	4.2 ± 3.2	6.0 ± 2.5[c,e]	3.5 ± 3.9[c,e]	10.4 ± 3.0[d,f]	2.9 ± 3.5[e]	12.9 ± 4.4[f]	2.6 ± 2.9
**Polyunsat. fat (g)**	1.8 ± 0.9[c,e]	1.0 ± 1.1[c,e]	5.0 ± 0.7[d,f]	0.7 ± 0.9[e]	5.9 ± 3.1[f]	0.9 ± 0.8	1.3 ± 0.8[c,e]	1.3 ± 1.5[c,e]	4.9 ± 1.8[d,f]	0.8 ± 1.4[e]	5.9 ± 2.5[f]	0.4 ± 0.4
**Monounsat. fat (g)**	3.7 ± 2.0[c,e]	1.9 ± 2.1[c,e]	13.3 ± 3.3[d,f]	2.2 ± 2.9[e]	14.3 ± 6.1[f]	3.1 ± 2.7	3.6 ± 1.6[c,e]	3.2 ± 3.4[c,e]	10.2 ± 3.2[d,f]	2.9 ± 5.2[e]	13.2 ± 2.9[f]	1.8 ± 1.6
**Fibre (g)**	6.0 ± 2.5[b,c,d,e,f]	1.4 ± 1.5[c,e]	9.5 ± 1.8[d,f]	1.0 ± 1.5[e]	8.5 ± 3.2[f]	1.0 ± 1.0	5.4 ± 2.2[b,d,e,f]	2.2 ± 3.0[c,e]	7.4 ± 1.1[d,f]	1.7 ± 1.8[e]	7.5 ± 2.0[f]	0.6 ± 0.4

Key: CHO, carbohydrate; DEI, daily energy intake; HCD, high carbohydrate diet; HFD, high fat diet. [1]Snacks are defined as any calorie consumption at least 15 min following the main meal; [2]Percentage relative to total energy from that mealtime. Data are mean ± SD; significantly different ($P<0.05$) within measure [b] compared to AM snacks, [c]compared to Lunch, [d] compared to PM snacks, [e] compared to Dinner, [f] compared to Late PM snacks.

Table S3. Anthropometric and resting energy expenditure measures from overweight, sedentary males (n=8) from baseline, the end of the habitual period, and the end of each experimental dietary period.

	Time of measure					
	**Baseline (Day -7)**		**Habitual (Day 0)**		**Experimental (Day 5)**	
	**Pre-HFD**	**Pre-HCD**	**Pre-HFD**	**Pre-HCD**	**HFD**	**HCD**
**Body composition**[1]						
**Body mass (kg)**	96.6 ± 10.1	96.9 ± 9.4	96.3 ± 9.2	96.6 ± 9.5	96.0 ± 9.3	96.1 ± 9/2
**Fat mass (kg)**	32.0 ± 4.9	31.6 ± 4.3	31.7 ± 4.8	31.4 ± 4.3	31.8 ± 4.8	31.3 ± 4.0
**Lean mass (kg)**	61.4 ± 7.3	62.0 ± 7.2	61.4 ± 6.8	61.9 ± 8.0	60.9 ± 6.9	62.1 ± 9.2
**Bone mineral content (kg)**	3.25 ± 0.47	3.29 ± 0.49	3.26 ± 0.46	3.26 ± 0.45	3.278± 0.47	3.26 ± 0.47
**Visceral adipose tissue (kg)**	1.74 ± 0.62	1.74 ± 0.62	1.75 ± 0.58	1.69 ± 0.49	1.70 ± 0.55	1.63 ± 0.57
**Body fat (%)**	34.2 ± 3.9	33.8 ± 3.8	34.0 ± 4.0	33.8 ± 4.2	34.3 ± 4.1	33.8 ± 3.7
**Resting energy expenditure (kJ/d)**	9056 ± 1047	9045 ± 1328	8806 ± 1069	9005 ± 1353	8759 ± 819	8504 ± 843

[1]From DXA measures; NS differences were observed between pre-condition measures.

Table S4. Wear time for activity monitor analysis across the habitual (Day -7 to Day 0) and Experimental (Day 1 – 5) periods for overweight, sedentary males (n=8).

	Time of measure			
	Habitual (Day -7 to 0)		Experimental (Day 1 to 5)	
	Pre-HFD	Pre-HCD	HFD	HCD
**ActivPal**, total wear time (min)[1]	970 ± 95	984 ± 103	955 ± 69	965 ± 80
**Actigraph**, wear time (min)[1]	950 ± 134	926 ± 166	938 ± 168	917 ± 145
Pre-prandial wear time, breakfast (min)	58 ± 3	58 ± 3	49 ± 14	51 ± 9
Pre-prandial wear time, lunch (min)	61 ± 1	60 ± 2	59 ± 3	60 ± 2
Pre-prandial wear time, dinner (min)	61 ± 1	60 ± 2	61 ± 0	60 ± 3
Postprandial wear time, breakfast (min)	176 ± 9	176 ± 10	169 ± 14	173 ± 12
Postprandial wear time, lunch (min)	176 ± 6	178 ± 4	175 ± 7	178 ± 4
Postprandial wear time, dinner (min)	163 ± 17	145 ± 29	164 ± 16	149 ± 21
**Sensewear**, wear time[2]				
Waking (h)	951 ± 246	932 ± 101	904 ± 232	966 ± 127
Sleep (h)[3]	469 ± 104	471 ± 103	488 ± 296	435 ± 115

[1]From Activpal and ActiGraph activity monitors where only waking hours were anlaysed, when total wake time was >10 h. [2]From SenseWear Armband monitors estimated from total wear time (>98%) over 24 h. [3]Data are n=7 due to one participant removing the monitor overnight for one condition. Data are mean ± SD.
